# Assessing the effect of virtual education on information literacy competency for evidence-based practice among the undergraduate nursing students

**DOI:** 10.1186/s12911-021-01418-9

**Published:** 2021-02-09

**Authors:** Maryam Shamsaee, Parvin Mangolian shahrbabaki, Leila Ahmadian, Jamileh Farokhzadian, Farhad Fatehi

**Affiliations:** 1 Department of Community Health Nursing, Kerman University of Medical Sciences, Kerman, Iran; 2 Nursing Research Center, Department of Critical Care Nursing, Razi Faculty of Nursing and Midwifery, Kerman University of Medical Sciences, Kerman, Iran; 3 Department of Health Information Sciences, Faculty of Management and Medical Information Sciences, Kerman University of Medical Sciences, Kerman, Iran; 4 Nursing Research Center, Kerman University of Medical Sciences, PO Box 7716913555, Haft-Bagh Highway, Kerman, Iran; 5 School of Psychological Sciences, Monash University, Melbourne, Australia; 6 Centre for Online Health, The University of Queensland, Brisbane, Australia

**Keywords:** Electronic-learning, Information literacy, Evidence-based nursing, Nursing students, Virtual learning

## Abstract

**Background:**

Information literacy competency is one of the requirements to implement Evidence-Based Practice (EBP) in nursing. It is necessary to pay attention to curricular development and use new educational methods such as virtual education to strengthen information literacy competency in nursing students. Given the scarcity of the studies on the effectiveness of virtual education in nursing, particularly in Iran, and the positive university atmosphere regarding the use of virtual education, this study investigated the effect of virtual education on the undergraduate nursing students’ information literacy competency for EBP.

**Methods:**

This interventional study was performed with two groups of intervention and control and a pretest and posttest design. Seventy-nine nursing students were selected and assigned to the intervention or control groups by random sampling. Virtual education of the information literacy was uploaded on a website in the form of six modules delivered in four weeks. Questionnaires of demographic information and information literacy for EBP were used to collect data before and one month after the virtual education.

**Results:**

The results showed no significant difference between the control and intervention groups in all dimensions of information literacy competency in the pre-test stage. In the post-test, the virtual education improved dimensions of information seeking skills (*t* = 3.14, *p* = 0.002) and knowledge about search operators (*t* = 39.84, *p* = 0.001) in the intervention groups compared with the control group. The virtual education did not have any significant effect on the use of different information resources and development of search strategy with assessing the frequency of selecting the most appropriate search statement in the intervention group.

**Conclusion:**

Virtual education had a significant effect on information seeking skills and knowledge about search operators in nursing students. Nurse educators can benefit from our experiences in designing this method for the use of virtual education programs in nursing schools. Given the lack of effectiveness of this program in using different information resources and development of search strategy, nurse educators are recommended to train information literacy for EBP by integrating several approaches such as virtual (online and offline) and face-to-face education.

## Background

Evidence-based practice (EBP) has been accepted as an important concept in nursing and is quickly becoming the norm for effective nursing practice globally [1]. EBP is a decision-making approach to patient care that integrates the most current and valid research findings, the nurse’s clinical expertise, the client's values and preferences, and available resources in making decisions. The benefits of EBP include nurses’ improved practical knowledge, patient-centered care with better patient outcomes, reduced occurrence of adverse events, reduced patient care costs, and health facility [2, 3].

Scientific evidence for EBP can be obtained by systematic and structured searches in retrieval systems, bibliographic databases, and clinical guidelines, which require retrieval skills in database searching and information literacy competency [2, 4]. Information literacy competency is the ability to recognize when information is needed, determine the amount of information needed, to retrieve information efficiently, and to evaluate, classify, and store information sources [5]. Information literacy competency is the ability to develop appropriate research questions, perform a search, appraise the relevant literature, and evaluate the transferability of research evidence into clinical practice and we know that it is critical to apply EBP successfully [6, 7].

Establishing information literacy competency in nursing students is vital for the promotion of EBP [8]. Nurse educators should develop nursing students’ competencies for EBP and then motivate them to deliver the highest quality of care using EBP [9]. Applying the effective strategies for teaching EBP competencies in theory and practice, they also have a fundamental role in developing information literacy competency among students [3]. Unfortunately, efforts to raise nursing students’ information literacy in the past years have had minimal success because experts and educators could not agree on effective strategies for nursing education and teaching of nursing information literacy [10]. Consequently, there are gaps in the integration of information literacy competency into nursing education [11]. In some studies, nursing students and newly graduated nurses reported that the training they received in nursing schools were insufficient, and they were not competent in almost all areas of informatics. These studies suggested that information literacy competency had to be improved through informatics curricula [11–13]. Moreover, researchers have reported that nursing schools do not have a standard for the type and complexity of computer skills required for nursing students. [14]. They also reported that factors and barriers affecting a student’s information literacy competency including, lack of information literacy in curriculum, confusing definitions of informatics, and nurse educators’ lack of informatics skill all might contribute to the student’s competency [13]. A systematic review reported that a lack of critical appraisal and advanced literature search skills might contribute to the negative attitudes towards EBP, which can be minimized by appropriate teaching of these skills [6].

It is noteworthy that there is an ongoing transition from traditional teaching and learning to more self-directed learning in nursing education due to the development of the Internet, learning platforms, and new technology [15] and change of the student population to digital natives. However, researchers have paid less attention to teaching strategies such as online teaching/learning and blended-learning about information literacy competency in Asian countries [10]. In addition, we are entering into a new phase of the evolution in academia and higher education known as “online and digital universities”. Digitalization in higher education allows streaming lectures online or enables professors and students to interact through virtual education [16]. Virtual education is a novel educational approach, which can facilitate simple and inexpensive access to educational resources and services through communication technologies (e.g., electronic devices) regardless of time and place [17].

Literature review showed that few studies implemented a training program to improve information literacy competency among students and reported that students’ information literacy competency increased after a training program. For example, Liou et al. tried to develop a blended course to improve nursing students’ information literacy competency in Taiwan. The majority of students with a positive perception of the teaching strategies expressed that they understood more about information literacy and applied information literacy skills in nursing [10]. A meta-analysis in Iran showed that virtual education was as effective as traditional education. Among different educational technologies, multimedia education, e-learning, and computer-mediated learning had significant effects on medical education. The educational technologies were more effective in the virtual education groups compared with the controls without virtual education [17]. Another study proposed various strategies for the incorporation of EBP competencies into undergraduate nursing education. Nurse educators can use strategies such as debates, social media, simulations, learning modules, game-based learning, workshops and training sessions. None of these strategies are superior to others in terms of teaching EBP [3].

## Problem statement

Health care in Iran has markedly improved over the last 25 years. The Iranian health care system aims to be evidence-based and patient-centered, which requires ongoing improvement of the quality of health care professional education. Nursing students need to be prepared for EBP competencies. Insufficient attention has been paid to teaching of information literacy competency in virtual nursing education, especially in middle- and low-income countries. Furthermore, virtual learning strengthens the traditional approaches to education, but there is no evidence of the evaluation of this method. Assessment of the students’ information literacy competency for EBP after virtual education can help adjust curricula to students’ educational needs. Regarding the importance this issue and the scarcity of the related studies in Iran, this study aimed to evaluate the impact of a training program on information literacy for EBP among the undergraduate nursing students in Iran.

## Methods

### Study design and setting

This interventional study with a pretest–posttest design was conducted in a Nursing School affiliated with Kerman University of Medical Sciences in southeast of Iran.

The bachelor's degree in Iran includes theoretical and practical courses in 8 semesters over four years in the university and educational hospitals. The undergraduate nursing curricula at the time of this study included 1.5 credits of research in nursing (34 h in third semester) and one credit of information technology in nursing (26 h in the first semester). According to the current curricula, the participants of this study did not receive educations related to information literacy competency.

### Study population and sampling

**Fig. 1:**
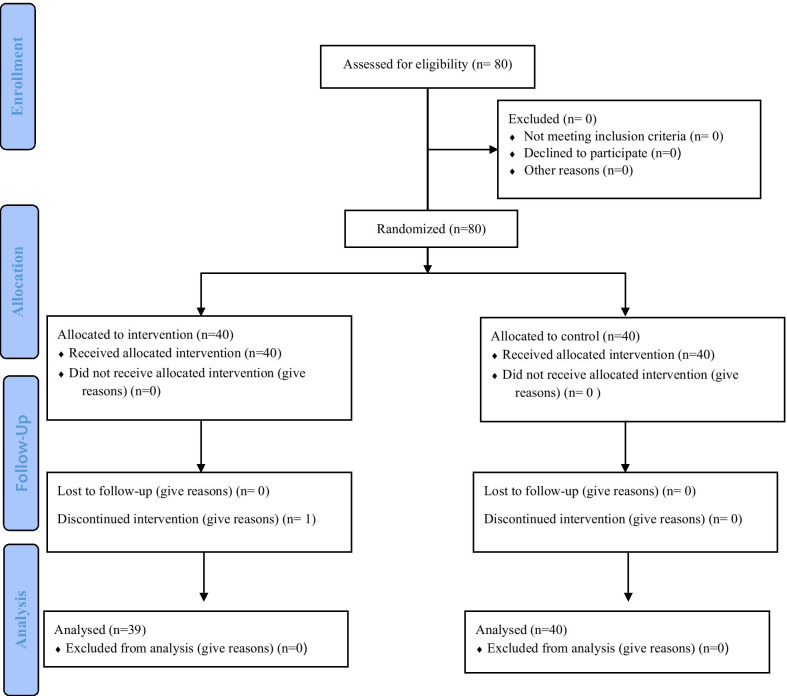
Flow diagram of the study, representing data collection points for the intervention group and the comparison group

The target population of this study included all undergraduate nursing students (N = 136) in the sixth and eighth semesters. The sample size was 80 participants by using the sample size formula that were divided randomly into intervention and control groups (40 students in each group) and equally selected from each of the semesters. Inclusion criteria included the nursing students who passed credits of research in nursing and information technology in nursing and started learning in the clinical settings. The students who were transferred to another university or failed to complete the questionnaires for any reason and guest students were excluded. Finally, 79 students completed the questionnaires and one student of the intervention group did not complete the course (response rate = 98.75%), (Fig. 1).

### Instruments

The instrument used in this study consisted of two questionnaires. The first one was about the nursing students' demographic information such as gender, age, so on (Table 2).

The second questionnaire was about information literacy competency for EBP, which was part of a questionnaire namely “Perceptions of Nurses of EBP” and was developed in the previous studies [18, 19]. This questionnaire composed of two sections. The first section concerned about the use of different information resources by nurses, including electronic, printed, and human sources (19 items). These items could be answered on a 5-point Likert scale ranging from "never" to "always". The second section included information searching skills and use of different search features of online databases and web search engines (10 items) on a 5-point Likert scale ranging from "never" to "always". In addition, nurses’ knowledge about Boolean /Connectors (‘OR ‘, AND’, ‘NOT’ or ‘AND NOT) and Proximity (e.g. W/nn; PRE/nn) operators was assessed. These items could be answered with yes (one point), no (zero point), and not sure (zero point). Finally, nurses were provided with a hypothetical searching topic (Effect of cigarettes on lung cancer) along with five possible search statements. They were asked to search MEDLINE using Boolean operators and select the most appropriate search statement for the given topic. Item 4 was the most appropriate search statement among the 5 available items (Cigarettes OR Smoking OR Tobacco) AND (“Lung Cancer” OR “Lung Tumor” OR “Lung Neoplasm”).

We used the Persian version of the questionnaire in this study, which was validated in Iran. The content validity and reliability (α = 0.87) of the Persian version of questionnaire was approved by experts [7].

### Data collection

Data were collected by using an anonymous, self-reported, and electronic questionnaire from March to May 2020. To collect data, the first researcher created groups for each of the intervention and control groups in WhatsApp. Informed consent was obtained from students enrolled in the course two weeks before the program. All students in this study had e-mail addresses; therefore, link of questionnaire was sent to e-mails and WhatsApp groups in pre-test (before course) and posttest stages (one month after the course). The instruction of how to fill out the questionnaires was sent to participants’ e-mails and WhatsApp groups. To attain the highest response rate, the first researcher spent appropriate time on data collection and determined a deadline to deliver completed questionnaires. In addition, she sent detailed information about how to learn the educational program. Moreover, she coordinated time of educational sessions with participants in the intervention group and reminded them to attend the sessions in the scheduled times. It should be noted that the participants completed questionnaires at e-campus.

### Intervention procedure

To prepare and develop the course content, the researchers reviewed the literature about information literacy competency and the link between information literacy and EBP in nursing [10, 20–24]. They discussed the extracted topics to achieve a consensus concerning goals and contents and teaching strategies. The researchers employed the standards proposed by the Association of College and Research Libraries as a guide for information literacy competency in higher education and selected essential competencies [24]. The team members provided their experiences and perspectives on required training of students concerning information literacy competency for EBP, as well as teaching–learning activities and educational programs available to informatics literacy in nursing school. This curricular integration also afforded many opportunities for student-centered teaching methods such as evidence-based learning, and inquiry learning. One medical informatics specialist and six nursing faculty members who were not in the research group approved the content validity of the educational content. Finally, two members, one specialized in nursing and the other in medical informatics, prepared topics of the course (Table 1).

**Table 1:** Topics presented in the modules and students’ participation rate in each educational modules

Modules	Topics	Missed (%)	Completed (%)
1	Familiarizing with EBP and understanding of what is involved in EBP Perceiving the value of EBP in nursing Learning the level of skills required for undertaking different EBP activities Discussing and making a possible work plan by using an example according to the steps of EBP Understanding information literacy and its framework Explaining various terminologies related to information literacy Familiarizing with how information technology can be used in education	1(2.50)	39(97.50)
2	Introducing and orientating variety of information sources including hard print, electronic and human sources Developing the skills to obtain e-books, e-journals, and other meaningful information using the library or the Internet Demonstrating a variety of electronic search capabilities such as the ways to subscribe and receive free articles Determining the most appropriate methods for accessing information electronically: search engines, interfaces (the database screens), and content available through a given system	2(5.00)	38(95.00)
3	Developing skills to criticize/evaluate hardware, software, and websites Identifying keywords, synonyms, and related terms for the information needed (Medline, etc.) Describing information needed through key concepts and terms Demonstrating medical and nursing databases such as Cumulative Index to Nursing & Allied Health Literature (CINAHL), PubMed, Scopus,… Using search strategies in databases such as PubMed and Scopus Introducing Up to Date, EBSCO,… Searching articles in Persian databases such as Scientific Information Database)SID(, Medlib, Iranmedex, and Magiran	1(2.50)	39(97.50)
4	Doing simple and advanced search, and conducting limited search based on the publication year, full text, keywords, Medical Subject Headings (MeSH), and using search operators such as AND, OR, NOT and etc Doing practical exercises. For example, retrieving related articles in databases such as PubMed and Scopus for “Intubate Patient Care” with related keywords and providing search results	3(7.50)	37(92.50)
5	Demonstrating abilities and gaining proficiency in search of information, management of information, and application of various technological tools in presenting information Determining the nature and extent of the information needed Explaining the risks and constraints of searching the Internet for needed evidence-based information Using appropriate search language and parameters for selected system	3(7.50)	37(92.50)
6	Assessing the quantity, quality, and relevance of the search results to determine whether alternative information retrieval systems or investigative methods should be utilized Evaluating information sources critically and incorporating selected information into their knowledge base and value system Comparing various information sources to evaluate reliability, validity, accuracy, authority, timeliness, and point of view or bias Synthesizing conclusions based upon information gathered Using information effectively for a specific purpose individually or as a member of a team Evaluating outcomes of the use of information	1(2.50)	39(97.50)

The educational materials were uploaded on the website dedicated to this research in form of six modules during four weeks. The participants of the intervention group had a username and password to use the educational content uploaded on the website. The address of the educational website was declared through the communication channels. They were able to access the website off line at any time and place. Reminder messages were sent via WhatsApp and SMS to motivate the use of the website. The communication between students and materials were prepared in the forms of audio file, PowerPoint slides, video tutorials, and textual help, question and answer, hands-on exercise (with examples of literature search), and homework. Students were required to self-study course materials, practice exercises, and discuss issues by E-mail or WhatsApp. Assignments included learning journals and literature search, criticizing website, discussing literacy issues such as academic integrity, and doing a search-based project. Assignments encouraged a sense of involvement in the use of reference materials. All other assignments were submitted to the instructors and their feedbacks were sent to the students via E-mail. The assignments could be resubmitted unlimitedly. While intervention group was provided with additional materials derived from our training course, the control group did not receive this program.

### Statistical analysis

The data were analyzed by using SPSS 21, descriptive statistics (frequency, percentage, mean and standard deviation) and inferential statistics (independent samples *t*-test, paired *t*-test, McNemar-test, and chi square). The Kolmogorov–Smirnov test showed that the data followed a normal distribution. The significance level was considered ≤ 0.05.

## Results

### Demographic and professional information

Table 2 shows demographic and professional information of the study participants. Based on the chi-square test, no significant difference was found between the intervention and control groups in demographic and professional information (Table 2). Data analysis indicated homogeneity of the participants in the two study groups at the baseline in all dimensions of the information literacy for EBP.

**Table 2:** Comparison of demographic and professional information of the nursing students between the intervention and control groups

Variables	Groups	Intervention		Control		Statistic)*χ2(*	*p-* value
	categories	n	%	n	%		
Gender	Male	18	64.20	27	67.50	3.60	0.06
	Female	21	53.80	13	32.50		
Marital status	Single	28	71.80	34	85.00	2.03	0.15
	Married	11	28.20	6	15.00		
Work experience in clinical setting (yr)	None	15	38.50	18	45.00	1.10	0.57
	Student work	24	61.50	22	55.00		
Attendance at nursing research courses	Yes	16	42.10	10	25	2.56	0.11
	No	23	57.90	30	75		
Attendance at computer skills courses	Yes	11	28.20	12	30.00	0.03	0.86
	No	28	71.80	28	70.00		
Attendance at information literacy courses	Yes	7	17.90	7	17.50	0.003	0.95
	No	32	82.10	33	82.50		
Willingness to use electronic databases and electronic journals	Low	6	15.80	12	32.40	3.19	0.20
	Moderate	18	47.40	16	43.20		
	High	14	36.80	9	24.30		

### Use of different information resources

According to Table 3, the pretest phase showed no significant difference in the mean scores of use of different information resources between the intervention (2.70 ± 0.57) and control (2.63 ± 0.44) groups (*t* = 1.39, *p* = 0.17). The results also showed that the control and intervention groups rarely used the electronic information resources with no change at posttest compared with pretest. However, no significant difference was observed between the intervention (2.53 ± 0.54) and control (2.71 ± 0.60) groups in the use of different information resources in the posttest (*t* = 1.33, *p* = 0.18). In addition, the paired *t*-test showed no significant change in the use of different information resources between the intervention and control groups at posttest compared with pretest.

**Table 3:** Comparison of the mean scores of the use of different information resources for patient care and clinical decision-making between intervention and control groups at pretest and posttest

Information resources	Time	Pretest	Posttest	Mean difference	Statistic t^a^ & p
	Groups	M ± SD	M ± SD		
Printed	Intervention	2.57 ± 0.64	2.64 ± 1.10	0.07	t = 0.96 p = 0.34
	Control	2.70 ± 0.72	2.60 ± 0.49	− 0.10	t = 0.73 p = 0.47
	Statistic t^b^ & p	t = 0.81 P = 0.42	t = 0.30 p = 0.76		
Electronic	Intervention	2.36 ± 0.73	2.27 ± 0.74	− 0.09	t = 0.88 p = 0.38
	Control	2.56 ± 0.75	2.51 ± 0.59	− 0.05	t = 0.40 p = 0.69
	Statistic t^b^ & p	t = 1.19 p = 0.23	t = 1.60 P = 0.11		
Human	Intervention	2.66 ± 0.52	2.62 ± 0.92	− 0.04	t = 0.25 p = 0.80
	Control	2.87 ± 0.68	2.84 ± 0.65	− 0.03	t = 0.25 p = 0.80
	Statistic t^b^ & p	t = 1.50 p = 0.14	t = 1.54 p = 0.13		
Total	Intervention	2.70 ± 0.57	2.53 ± 0.54	− 0.17	t = 0.53 P = 0.60
	Control	2.63 ± 0.44	2.71 ± 0.60	0.08	t = 0.48 p = 0.63
	Statistic t^b^ & p	t = 1.39 P = 0.17	t = 1.33 P = 0.18		

^a^Paired *t*-test

^b^Independent *t*-test

### Information searching skills

The pretest phase showed no significant difference in the mean scores of information searching skills and use of the different search features between the intervention (2.33 ± 0.74) and control (2.45 ± 0.64) groups (*t* = 0.68, *p* = *0.50*). However, a significant difference was observed between the intervention (2.58 ± 0.31) and control (2.17 ± 0.58) groups in terms of information searching skills and use of the different search features in the posttest (*t* = 3.14, *p* = 0.002). In addition, the paired *t*-test showed that searching skills and use of the different search features statistically significantly decreased in the control group in posttest compared with pretest (Table 4).

**Table 4:** Comparison of the mean scores of information searching skills and the use of different search features between intervention and control groups at pre- and posttest

variable	Time	Pre test	Post test	Mean difference	Statistic t^a^ & p
	Groups	M ± SD	M ± SD		
Information searching skills	Intervention	2.33 ± 0.74	2.85 ± 0.31	0.52	t = 2.40 p = 0.02
	Control	2.45 ± 0.64	2.17 ± 0.58	− 0.28	t = 2.36 p = 0.02
	Statistic t^b^ & p	t = 0.68 p = 0.50	t = 3.14 p = 0.002		

^a^Paired *t*-test

^b^Independent *t*-test

### Knowledge about search operators

The pretest phase showed no significant difference between the intervention (0.37 ± 0.09) and control (0.25 ± 0.08) groups in the mean scores of knowledge about search operators (*t* = 1.54, *p* = 0.12). However, a statistically significant improvement was observed in the intervention group (0.67 ± 0.07) compared with the control group (0.34 ± 0.17) in terms of knowledge about search operators in the posttest (*t* = 8.39, *p* = 0.001). In addition, the paired *t-*test showed no significant difference in the control group’s knowledge about search operators at posttest compared with pretest (Table 5).

**Table 5:** Comparison of mean scores of knowledge about search operators between intervention and control groups at pre- and posttest

Variable	Time Groups	Posttest	Pretest	Mean difference	Statistic t^a^ &p
		M ± SD	M ± SD		
Knowledge about search operators	Intervention	0.37 ± 0.09	0.67 ± 0.07	0.30	t = 5.08 p = 0.001
	Control	0.25 ± 0.08	0.34 ± 0.17	0.09	t = 1.21 p = 0.23
	Statistic t^b^ & p	t = 1.54 p = 0. 12	t = 8.39 p = 0. 001		

^a^Paired *t*-test

^b^Independent *t*-test

**Table 6:** Comparison of frequency of selecting the most appropriate search statement in intervention and control groups at pretest and posttest

Variable	Groups	Posttest	Pretest	Statistic^a^ &
		n (%)	n (%)	
Selecting the most appropriate search statement	Intervention	16 (41)	16 (41)	P = 1.10
	Control	10 (25)	8 (20)	
	Statistic^b^ & p	X^2^ = 2.30, p = 0.13	X^2 ^= 4.12, p = 0.04	p = 0.62

^a^Mc Nemar-test

^b^chi square test

### Assessing development of search strategy with assessing the frequency of selecting the most appropriate search statement

The students were given a supposed subject “Effect of cigarettes on lung cancer” to assess their skills in developing an effective search statement by using Boolean operators. For simplicity, the certain search features, such as truncations, proximity operators and extensive synonyms were avoided. The students were asked to choose the most appropriate statement among five possible search statements. The pretest phase showed no significant difference between the intervention (41%, n = 16) and control (25%, n = 10) groups in frequency of selecting the most appropriate search statement (*X*^*2*^ = 2.30, *p* = 0.13). In the posttest, the frequency of selecting the most appropriate search statement (41%, n = 16) did not change in the intervention group, but it significantly decreased in the control group compared with intervention group (20%, n = 8), (*X*^*2*^ = 4.12, *p* = 0.04). In addition, the McNemar-test showed that frequency of selecting the most appropriate search statement had no significant change in the control group at posttest compared with pretest (Table 6).

## Discussion

This study evaluated the effect of virtual education on the undergraduate nursing students’ information literacy competency for EBP. The results showed that the educational program did not improve significantly the use of different information resources and its dimensions (printed, electronic, and human resources) in the students of the intervention group. The researchers did not find similar interventional studies whose results support the present study. However, several studies [9, 25–28] showed a positive effect of the educational interventions on the use of information resources.

Moreover, the results showed that the control and intervention groups rarely used the electronic information resources, and this educational program could not motivate students in the intervention group to use electronic resources more to search for information on clinical decision-making and EBP. However, a study showed that electronic resources were the most important sources of information for providing health care among postgraduate students [29].

In agreement with these results, researchers in previous studies [7, 30–32] reported that nurses considered their coworkers more efficient than printed information sources because they were more accessible to them. They also believed that human resources would give them an opportunity to discuss clinical decisions and they would gain more confidence [32]. Onyia considered the high cost of electronic resources and Internet access, insufficient library equipment, as well as Internet speed as the reasons for poor use of online resources [31]. In a systematic review, the main barriers reported by nurses related to seeking Internet information were lack of time to search the web, insufficient skills in searching and retrieving information, inability to translate English texts, and the unawareness from the library as a reliable and efficient place for seeking on-line information. Nurses acknowledged lack of appropriate technology infrastructure, little technical and nursing management support, and improper physical location of computers as other barriers of the use of the electronic information resources. Moreover, nurses relied more on their implicit and traditional knowledge during the process of searching and evaluation of clinical guidelines, which is considered risky because it might result in an inadequate and unsafe practice [32].

The results showed that the educational program increased searching skills and use of different search features in the intervention group compared with the control group. Previous studies also have shown the effectiveness of educational interventions in the posttest and improvement of searching skills in participants [9, 33–37]. A literature review reported that nurses were more confident in using Google than using bibliographic databases. PubMed or MEDLINE and CINAHL were the most commonly used bibliographic databases for information retrieval. Although there is an overall global increase in the use of electronic devices, such as computers, mobile devices and smartphones, and much information is available to the Internet or databases, the nurses’ use of bibliographic databases is significantly low. Nurses still prefer searching information on resources such as Google, as well as consulting and asking coworkers rather than searching bibliographic databases [4].

According to our results, the educational program had a positive effect on the intervention group’s knowledge of using Boolean and proximity operators compared with the control group. Several descriptive studies on the use of operators showed the weakness of users [18, 38]. In agreement with our results, a study showed that students’ knowledge about search statement and Boolean operator increased after educational course [36]. Owing to the fact that information retrieval systems, including databases and search engines hold millions of records, students should learn correct and advance search, controlled terms, synonyms and terms related to words using Boolean, proximity operators and other search strategies.

This study showed no improvement in the ability of the intervention group after the educational program to develop an online search strategy with selecting the most appropriate search statement. Several studies reported that few nurses selected the most appropriate search statement of the questionnaire using some synonyms of the concepts in a given subject. It should be noted that the questionnaire of the present study was derived from these studies [7, 18, 19]. In agreement with our results, a study implemented an online information literacy tutorial and a face-to-face session for teaching information literacy competency. The results showed that searching skills, including development of search strategy and use of search operators remained unchanged in both method one month later [35]. However, another study reported improvement in the search of information resources and search strategy among nursing master’s students through an educational program [39].

The results showed that searching skills and use of different search features statistically significantly decreased in the control group at posttest compared with pretest. Researchers believe that various factors can contribute to the low score of control groups, and the ineffectiveness of this program in the use of different information resources and development of search strategy in intervention group. For example, the educational program was done at the same time as the COVID-19 pandemic. The multiple stressors caused by the COVID-19, the conditions governing the country, the closure of the university, life in quarantine and the unpreparedness of students for this educational method also might contribute to the ineffectiveness of intervention, and we did not examine the impact of them in this study. In addition, readiness, educational backgrounds and practical experiences may affect our results. According to researchers in a study, although digitalization in higher education allows streaming lectures online or enables professors and students to interact in the virtual environments, not everyone is ready for digitalization. Even those young people, who spend much time on playing video games or interacting with others on social network platforms, prefer real classrooms and universities [16]. Other reasons include inadequate skills of professors in using electronic resources, and the age gap between educators (as Digital Immigrants) and nursing students (as native Immigrants) [40]. Educators have challenges with a changing student population, who are mostly younger than 25 years of age and are considered as “digital natives”. They respond to information technology much more sufficiently and effectively and their desire to learn with interactive means is stronger than that of the “old scholar” (equal or older than 25 years old). Therefore, innovative teaching strategies are needed in teaching and learning to integrate information literacy into nursing curriculum [10].

In total, the results of this study may be somewhat similar to other studies. However, the discrepancy between some of our results and the abovementioned studies can be due to the differences in community, study population, sampling, randomization and matching of the groups, educational backgrounds and students’ familiarity with computers, educational content and method, lack of face-to-face interaction with participants in virtual education, data collection tools, conditions, study setting and time, and nurse educator preparation levels. For example, participants in our study were undergraduate students nursing. In Iran, nursing students are unfamiliar with EBP and have poor understanding of EBP and the importance of using information literacy in EBP. No student is involved in EBP system due to inefficiency of the educational system in the university. Moreover, nurse instructors do not have a proper understanding of EBP and therefore do not emphasize on EBP prerequisites.

Finally, the researchers in this study believe that they cannot achieve effective education by only presentation of the virtual content. It is impossible to achieve educational goals without paying attention to the educational design and models in various formats. Therefore, nurse instructors should achieve effective e-learning by expanding the use of educational design patterns, developing electronic courses and using effective models for improvement of nursing students’ information literacy competency for EBP.

## Limitations

This study had three limitations. First, the study was conducted during the COVID-19 pandemic within a single setting affiliated with a medical university in Iran. We did not have any knowledge about evaluation of information literacy for EBP in students before this time for comparison. Second, we did not assess whether the additional training for information literacy had a detrimental effect on other aspects of nursing education. In this study, we only measured the effectiveness of the educational program with self-report tool and did not evaluate the speed and skills of nursing in practice when developing a search strategy and finding the appropriate resources. Future study can address and evaluate the effectiveness of such educational program in nursing practice. Third, data collection was conducted one month after the intervention. The 3–6 month follow-ups or the post-pandemic follow-up are recommended to have results that are more accurate and to determine the long-term impact of training. Different teaching methods such as online teaching can be applied to determine the best method of teaching information literacy for EBP. Future longitudinal and interventional studies can be performed by using blended methods of education and evaluation of information literacy competency to help determine the actual competency.

## Conclusions

According to the results, virtual education had a positive effect on two aspects of information literacy competency, including information seeking skills and knowledge about search operators in intervention group. The virtual education did not have a positive effect on aspects of the use of information resources and development of search strategy as reported by the students. Future study could evaluate these skills in practice to have better insight regarding the effectiveness of the educational program. However, the use of virtual education and computer-based learning modules, which are cost-effective and offer several benefits in medical education, may not necessarily be the most effective methods for teaching information literacy competency for EBP. Nursing professors and instructors are suggested to combine virtual (online and offline) and face-to-face methods according to conditions and the needs of students to develop educational syllabus in the field of information literacy for EBP and coordinate educational materials in theory and practice. Our experiences in designing this program could be beneficial for developing future educational programs aimed at fostering competencies in information literacy and EBP by nurse educators.

## Data Availability

The data are available upon request to the corresponding author after signing appropriate documents in line with ethical application and the decision of the Ethics Committee.

## Abbreviations

CINA HL: Cumulative Index to Nursing & Allied Health Literature
EBP: Evidence-based practice
MeSH: Medical Subject Headings
SID: Scientific Information Database
